# Urinary Congophilia in Pre‐Eclampsia: A Potential Diagnostic Biomarker

**DOI:** 10.1155/jp/6496909

**Published:** 2026-06-30

**Authors:** Mikyle David, Olive Khaliq, Anand Krishnan, Niren Maharaj

**Affiliations:** ^1^ Department of Obstetrics and Gynaecology, School of Clinical Medicine, Faculty of Health Sciences, University of the Free State, Bloemfontein, South Africa, ufs.ac.za; ^2^ Department of Paediatrics and Child Health, School of Clinical Medicine, Faculty of Health Sciences, University of the Free State, Bloemfontein, South Africa, ufs.ac.za; ^3^ Department of Haematology and Cell Biology, School of Pathology, Faculty of Health Sciences, University of the Free State, Bloemfontein, South Africa, ufs.ac.za

**Keywords:** Congo red dot test, diagnostic biomarkers, low-resource settings, pre-eclampsia, urinary congophilia

## Abstract

**Aim:**

Pre‐eclampsia (PE) causes significant maternal and neonatal morbidity and mortality worldwide, particularly in low‐ and middle‐income countries (LMICs). Current diagnostic methods, such as blood pressure monitoring and proteinuria measurement, have poor sensitivity and specificity. Although biomarkers like PlGF and sFlt‐1 improve diagnostic accuracy, their clinical value is limited by cost and infrastructural constraints. The Congo red dot (CRD) test, which identifies misfolded proteins via urinary congophilia, has emerged as a low‐cost, noninvasive alternative for detecting PE.

**Methods:**

This review evaluated the diagnostic performance of the CRD test by synthesizing evidence from studies across diverse populations, including India, China, and South Africa. Methodologies encompassed prospective cohorts, case–control designs, and meta‐analyses, with a focus on sensitivity, specificity, and comparative efficacy against conventional screening tools.

**Results:**

Results indicate that the CRD test achieves high diagnostic accuracy, with reported specificity of 89.2% and sensitivity of 80.2%. The assay provides rapid results (< 3 min) without specialized equipment, demonstrating effectiveness in LMICs. However, performance variability was observed in late‐onset PE, and comparative studies yielded conflicting data regarding its superiority over urinary dipstick analysis. Additionally, challenges persist in differentiating PE from other hypertensive disorders with overlapping proteinuria.

**Conclusion:**

The CRD test represents a promising diagnostic tool for PE in LMICS with limited access to advanced diagnostics. Further large‐scale validation studies are required to standardize protocols, establish gestational age–specific thresholds, and clarify its role in differentiating PE from confounding conditions. Integration with existing biomarkers may enhance its clinical adoption and diagnostic precision.

## 1. Introduction

Pre‐eclampsia (PE) remains a significant cause of maternal and perinatal morbidity and mortality globally, accounting for an estimated 76,000 maternal and 500,000 perinatal deaths annually [1]. As its prevalence rises worldwide, particularly in low‐ and middle‐income countries (LMICs), the need for accurate diagnostic and prediction methods becomes critical [2]. PE, which causes new‐onset hypertension and organ failure after 20 weeks of gestation, has a wide range of clinical presentations, from minor symptoms to severe consequences such as eclampsia, intrauterine growth restriction (IUGR), and maternal end‐organ damage [1, 3, 4]. Despite significant breakthroughs in antenatal care (ANC), diagnostic delays continue, frequently due to a lack of effective, accessible, and timely biomarkers.

Traditional diagnostic criteria based on blood pressure and proteinuria are often insufficient for early diagnosis or separation from other hypertensive diseases of pregnancy (HDP) [1, 4]. Although blood‐based biomarkers like placental growth factor (PlGF) and soluble fms‐like tyrosine kinase‐1 (sFlt‐1) improve diagnostic accuracy, their high cost and infrastructure constraints prevent widespread use in LMICs. As a result, there is growing interest in innovative, low‐cost, noninvasive biomarkers capable of accurately predicting and diagnosing PE, particularly in resource‐constrained situations [5].

Recent research has revealed protein misfolding and amyloid‐like clumps in the urine of women with PE, adding to our understanding of its pathogenesis [6]. Misfolded proteins, which were formerly linked to neurodegenerative and inflammatory illnesses such as Alzheimer’s, are now recognized to occur in systemic and oxidative stress circumstances, such as those found in PE [7, 8]. Congo red dye can identify misfolded proteins in urine by binding to *β*‐sheet‐rich structures found in amyloid fibrils [9]. This interaction underpins the Congo red dot (CRD) test, a paper‐based assay that visually detects congophilia in urine samples.

Several studies have validated the CRD test’s utility in PE diagnosis, highlighting its high specificity, cost‐effectiveness, and quick turnaround time, indicating Congo red staining’s promise as both a diagnostic and prognostic tool (Table 1). The Congo red assay’s noninvasiveness, simplicity, and low cost make it ideal for application in LMICs with limited access to advanced diagnostics. Studies from India, China, and South Africa have yielded promising outcomes, but data from other high‐burden locations, such as sub‐Saharan Africa, are lacking. Future clinical validation studies, particularly multicenter trials, are critical for establishing clinical value, standardizing techniques, and obtaining regulatory approval.

**Table 1:** Summary of studies that explored the use of the Congo red dye to predict pre‐eclampsia.

Study (year)	Country	Design	Sample size	GA (weeks)	Index test	Comparator	Performance	Limitation
Sailakshmi et al. (2021) [10]	India	Prospective	NR	≥ 20	CRD	Clinical PE	High accuracy	Single center
Tuntivararut et al. (2024) [11]	Thailand	Prospective MC	244	≥ 20	CRD ≥ 4	Clinical PE	Se 49.6%; Sp 94.7%	↓ sensitivity
Khaliq et al. (2023) [9]	South Africa	SR/MA	17 studies	Var	CRD	Standard PE	Inconclusive	Heterogeneity
Bracken et al. (2020) [12]	BD/MX	Case–control	516	≥ 20	CRD	BP + proteinuria	Se 83%; Sp 87%	Study design
Rood et al. (2019) [13]	United States	Prospective	346	20–37	CRD	ACOG	AUC 0.89	No subtyping
Fedotov et al. (2023) [14]	Russia	Case–control	168	≥ 20	CRD	CKD	↓ specificity	Renal bias
Petca et al. (2022) [15]	Romania	Review	NR	Var	CRD	Standard PE	Supportive	No data
Rani et al. (2022) [16]	India	Prospective	NR	≥ 20	CRD	Clinical PE	Early signal	Small sample size
Döbert et al. (2022) [17]	United Kingdom	Prospective	2140	35–37	CRD	ISSHP	Se < 10*%*	LO‐PE only
Wong et al. (2023) [18]	Hong Kong	Prospective	NR	≥ 20	CRD	Clinical PE	↑ Sp; ↓ Se	Short window
Sharma et al. (2024) [19]	India	Prospective	NR	≥ 20	CRD	Clinical PE	High NPV	No follow‐up
Gupta et al. (2024) [20]	India	Case–control	NR	≥ 20	CRD vs. UV	UV–Vis	UV superior	Feasibility
Liu et al. (2024) [21]	China	Prospective	NR	≥ 20	CercaTest	Clinical PE	High accuracy	Single site
Chávez et al. (2018) [21]	United Kingdom	Transversal	NR	≥ 20	CR stain	Proteinuria	High selectivity	Cross‐sectional
Wang et al. (2024) [22]	China	Prospective	409	≥ 20	CRD vs. dipstick	Dipstick	Sp 90% vs. Se 77%	No proteomics
Anwar et al. (2022) [23]	Pakistan	Review	NR	Var	CRD	Standard PE	Promising	No data
Cai et al. (2021) [24]	China	Retrospective	1397	< 34	CapCord	HDP	↑ EO‐PE signal	Retrospective

Abbreviations: AUC, area under ROC curve; CKD, chronic kidney disease; CRD, Congo red dot; EO‐PE, early‐onset PE; GA, gestational age; LO‐PE, late‐onset PE; MC, multicenter; NPV, negative predictive value; NR, not reported; PE, pre‐eclampsia; Se, sensitivity; Sp, specificity; SR/MA, systematic review/meta‐analysis; Var, variable.

This review is aimed at synthesizing current literature on Congo Red Dye and exploring its potential role as a noninvasive biomarker for PE.

## 2. Classification of PE

While most PE cases (80%) occur after 37 weeks of pregnancy (late‐onset or term PE), early‐onset or “preterm PE” occurs less commonly between 20 and 34 weeks of pregnancy and is associated with more complications. These include low birth weight, fetal growth restriction, preterm birth, stillbirth, or neonatal mortality, as well as severe maternal morbidity and death [25]. The current treatment for PE and its complications is the delivery of the fetus and placenta. Approximately 60% of normotensive pregnant women develop postpartum PE, which causes new‐onset hypertension between 48 h and 6 weeks after birth, typically between 7 and 10 days. Postpartum PE is not widely recognized, although risk factors include older maternal age, obesity, ethnicity, and cesarean birth [26].

## 3. Pathogenesis of PE

**Figure 1:**
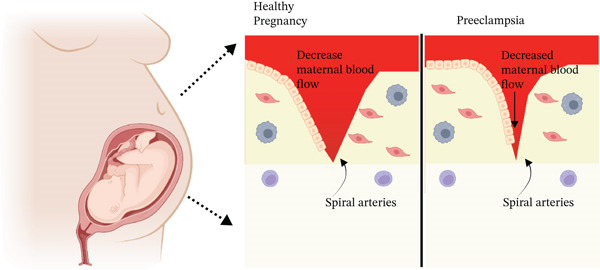
Schematic overview of the pathogenesis of pre‐eclampsia. Inadequate migration of extravillous trophoblast (EVT) cells results in shallow trophoblast invasion, reducing spiral artery remodelling and overall lack of nutrients to the fetus.

The pathophysiology of PE is not entirely elucidated; nonetheless, in PE, there is a defective migration of extravillous trophoblast (EVT) cells with nonphysiological conversion of the myometrial spiral arteries [27]. This reduces the diameter of blood vessels, resulting in an insufficient blood supply to satisfy the oxygen and nutrition requirements (Figure 1) for the fetus [28]. Furthermore, this causes hypoxia in the placenta, resulting in the release of antiangiogenic substances such as soluble endoglin, sFlt‐1, and inflammatory mediators into the maternal systemic circulation [29, 30].

## 4. Diagnostic and Treatment Approaches for PE

Pregnant women at risk of PE require high‐quality antenatal, intrapartum, and postpartum services. This includes thorough clinical and laboratory evaluations, continuous patient and healthcare provider communication, and a multidisciplinary team. Late initiation of ANC is a key barrier to preventing PE early, as many women do not attend their first visit in the first trimester [31]. This delay hinders timely risk identification and reduces the chance of implementing effective strategies to lower complications, prevent preterm delivery, and improve health outcomes for both mother and baby. Recent updates to PE diagnostic criteria and understanding genetic and environmental risk factors are increasing interest for researchers.

## 5. Current Screening Methods

Blood‐based biomarker testing has significantly improved the early detection and management of PE. PlGF and serum FMS‐like tyrosine kinase‐1 (sFlt‐1) are widely studied biomarkers for predicting PE onset and identifying cases likely to progress to severe disease [32, 33]. PlGF levels are typically reduced in PE patients, while sFlt‐1 concentrations are elevated in pregnancies complicated by PE after 20 weeks’ gestation. Glycosylated fibronectin (GlyFn) is also a promising candidate biomarker [34]. Professional societies like the International Federation of Gynecology and Obstetrics (FIGO) and the International Society for the Study of Hypertension in Pregnancy (ISSHP) endorse the clinical use of PlGF, sFlt‐1, and other biomarkers as part of a “rule‐out” approach for PE [35].

Several point‐of‐care (POC) and near‐POC diagnostic platforms measuring PlGF, sFlt‐1, and GlyFn have been developed, and some adapted for low‐resource settings. Evidence supports their validation in LMICs, with ongoing studies assessing their integration into clinical decision‐making algorithms and real‐world performance [4, 36]. Ongoing research and innovation enhance strategies for preventing and screening PE and building on high‐quality ANC. Routine blood pressure monitoring is central to diagnosing and assessing women at risk for PE. However, many ANC settings lack access to validated BP monitors and need improved training on accurate measurement techniques and interpretation [35, 37].

A new randomized controlled trial, PEARLS (Preventing pre‐eclampsia: Evaluating Aspirin Low‐dose regimens), is currently comparing the efficacy and safety of 75 and 150 mg of daily aspirin for women at elevated risk of PE. The trial is being conducted in Ghana, Kenya, and South Africa, aiming to address questions about the optimal dosing strategy for maximum preventive benefit [38]. Calcium supplementation, another recommended intervention for pregnant women, has demonstrated efficacy and safety in reducing PE risk, but its integration into routine ANC remains inconsistent across settings [36, 38]. Ultrasonography is also advised to accurately determine gestational age and screen for structural abnormalities, particularly in LMICs, to identify IUGR, a fetal complication commonly associated with PE [6].

## 6. Role of Urinary Biomarkers in PE

Efforts are underway to identify novel biomarkers for PE as both predictive and diagnostic tools. Biomarkers for PE primarily originate from organs involved in its etiology, such as the placenta, cardiovascular system, and kidneys. Angiogenic factors, including VEGF, PlGF, and their receptors sFlt‐1, play critical roles in placental angiogenesis and have been extensively studied in recent years [35]. Placental hypoxia and oxidative stress increase the production of placental‐derived factors, resulting in altered blood levels of sFlt‐1, VEGF, and PlGF in women with clinical PE. However, recurrent measurements of these molecules during regular prenatal care remain difficult. A viable and less invasive screening tool could be the detection of proteins in urine [39].

Collection and analysis of urine samples provide several benefits over blood samples when it comes to illness monitoring and diagnosis. Urine collection is a reasonably simple and noninvasive technique, which makes it easier for patients. Urine collection can be performed more frequently than blood collection since it causes less harm, enabling ongoing and sequential tracking of the development of disorders throughout time [40]. Urine can include a wide range of chemicals, proteins, and metabolites, which, when stored properly, show superior stability to plasma, which is another important factor [40]. This is advantageous, offering insights into the individual’s metabolic and physiological status, therefore facilitating the early diagnosis of renal, hepatic, metabolic, and infectious disorders and enabling the monitoring of chronic conditions, such as hypertension [41]. Urinary biomarkers have shown effectiveness and clinical utility in certain kidney‐related conditions, including Type 2 diabetes and systemic lupus erythematosus. However, their clinical use in PE remains quite limited, particularly in LMICs [32].

## 7. Potential for Proteomics and Metabolomics in PE Detection

Technological advancements in mass spectrometers have led to the growing use of proteomics and metabolomics. Large‐scale protein and metabolite analysis, known as proteomics and metabolomics, is becoming more common in kidney research, with studies using mass spectrometry to examine urine indicators in the setting of PE [7, 39, 42].

Multiple studies have explored urinary protein and metabolite profiles as predictive markers for PE. A 2011 investigation by Moreno‐Gonzalez and Soto [8] developed a predictive model comprising 50 urinary biomarkers, demonstrating 100% sensitivity and specificity for identifying the onset of PE at 28 weeks of gestation. More recently, in 2023, Varghese et al. [43] conducted a cohort study involving 60 pregnant women and proposed a novel urine‐based metabolomic signature. This seven‐compound panel showed strong potential in predicting PE as early as 8–20 weeks of gestation, highlighting its promise for early disease detection [40].

## 8. Misfolded Proteins and Urinary Congophilia in PE

In human cells, amino acids are assembled into long chains that fold into specific three‐dimensional shapes to become functional proteins. However, this precise folding process can go awry in certain disease states, resulting in misfolded or structurally abnormal proteins. These misfolded proteins may lose their normal function and become toxic to cells [7, 8]. While they are well known for their role in forming amyloid plaques in neurodegenerative conditions like Alzheimer’s and Parkinson’s disease, recent studies have found that misfolded proteins can also be detected in the urine, serum, and placental tissue of pregnant women with PE. The exact source of these misfolded proteins in the urine of women with PE is still not fully understood [39].

Misfolded proteins are reported to occur prior to the clinical presentations of PE, highlighting the importance of using Congo red dye to detect PE in women at risk. Congo red, a dye that binds specifically to *β*‐sheet structures in amyloid fibrils, a property known as congophilia, has long been used as a gold standard to identify these types of misfolded proteins [9]. Researchers begun investigating this unique binding property as a potential diagnostic marker for PE [44]. Early diagnosis and early intervention of PE in pregnancy are crucial for the development of the fetus as PE may lead to adverse outcomes such as low birth weight, stillbirths, IUGR, and death [28–30]. The use of congophilia for detecting PE is widely supported [10, 13, 17, 22, 44, 45].

In a groundbreaking study, Buhimschi et al. [42] found that urine from women with PE showed notable congophilia, particularly in cases of severe PE that required early delivery. This was not observed in healthy pregnancies or in women with chronic or gestational hypertension. Interestingly, research indicated that the test may distinguish PE from other hypertensive disorders of pregnancy, a current challenge, especially in low‐resource settings such as LMICs, using the Congo red dye paper test [45].

**Figure 2:**
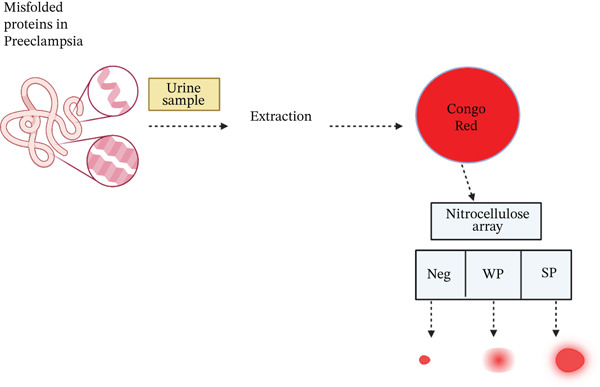
Schematic illustration of misfolded proteins in Congo red. Urine samples are collected, and proteins are extracted, followed by incubation with Congo Red dye, which binds specifically to misfolded protein aggregates. The mixture is applied to a nitrocellulose membrane, and signal intensity is used to classify results as negative (Neg), weak positive (WP), or strong positive (SP) based on the degree of Congo Red retention.

The test is reported to have high sensitivity (80.2%), specificity (89.2%), and accuracy (86.7%) [13]. The test is noninvasive, quick, and easy to use. Results can be viewed in approximately 3 min. No preprocessing steps are required, and midstream urine is used at room temperature. The dye in the test is used to bind to amyloid‐like proteins (misfolded proteins), while the dye binds to the cellulose fibers. A stain is formed on the nitrocellulose array; if a small stain appears, then the test is negative. If a bigger stain forms, less red in color, this is regarded as a weak positive (WP). In contrast, a larger deep red stain signifies a strong positive (SP) result, indicating that more proteins were available for the Congo red dye to bind to (Figure 2).

Various studies in LMICs explored the use of the CRD paper test to predict PE in women suspected to have the condition and those at risk of developing the condition. One study indicated that the mean Congo red retention was higher in women with PE compared to normotensive women (77.9 ± 11.5 vs. 37.9 ± 4.1) and concluded that the test was a good predictor with increased retention in both mild and severe PE [44]. Other studies reported similar findings [11, 13, 45]. However, contradictory results were reported in two other studies. One study reported that the CRD paper test was a poor predictor of PE in women 35–37 weeks of gestation, as some women without PE obtained a positive congophilia result [17]. Comparably, another study found that the urinary dipstick had a higher sensitivity and was more accurate in detecting proteinuria compared to the CRD paper test [22].

Table 1 summarizes the findings reported by various studies worldwide on the effectiveness of the Congo red dye in detecting misfolded proteins in women with PE. A total of 17 studies are included in the table, and 4 (23%) of the studies reported that Congo red dye is a poor predictor of PE. These studies concluded that other forms of prediction, such as the urinary dipstick analysis and the UV–Vis absorption spectroscopy, were better tools than the Congo red dye. In contrast, the other studies favored using the Congo red dye to predict PE early. The Congo red dye has been reported as accurate and cost‐effective by 76% of the studies with a high sensitivity and specificity. This table also shows that most studies were conducted in India and China, with limited studies in other countries like the United States, the United Kingdom, Russia, Bangladesh, and Mexico. One article was published in South Africa, which was a systematic review. However, this study included original articles from other countries with no data from South Africa, a country with a high burden of HDP. South Africa is a LMIC with limited resources and disadvantaged populations in need of medical attention, and it would benefit from the Congo red dye test.

## 9. Conclusions and Further Recommendations

The CRD test represents a promising, low‐cost, rapid, and noninvasive diagnostic tool for PE, particularly in LMICs where access to advanced biomarker assays may be limited. The test has a high sensitivity and specificity and the advantage of delivering results rapidly without the need for specialized equipment.

Although robust data regarding its superiority over conventional dipstick proteinuria testing is limited, emerging evidence suggests that the CRD test offers important mechanistic advantages by targeting misfolded proteins specific to the pathophysiology of PE rather than relying solely on nonspecific proteinuria. While differentiation between PE and other hypertensive disorders of pregnancy remains a challenge, the CRD assay holds potential to enhance clinical diagnosis when incorporated into structured clinical assessment and multimodal diagnostic strategies.

Future large‐scale, multicenter validation studies are essential to standardize testing protocols, establish gestational age–specific diagnostic thresholds, and clarify its comparative and complementary role alongside established biomarkers such as PlGF and sFlt‐1. Integration of the CRD test into multimodal diagnostic algorithms may enhance early detection, improve risk stratification, and ultimately contribute to better maternal and neonatal outcomes, particularly in resource‐constrained healthcare systems.

## Author Contributions

Mikyle David: manuscript writing, conduct, and data analysis. Olive Khaliq: planning, manuscript writing, and data analysis. Anand Krishnan: conceptualizing, design, and planning. Niren Maharaj: manuscript editing and supervision.

## Funding

No funding was received for this manuscript.

## Conflicts of Interest

The authors declare no conflicts of interest.

## Data Availability Statement

Data sharing is not applicable to this article as no datasets were generated or analyzed during the current study.
